# Enhancer remodeling activates NOTCH3 signaling to confer chemoresistance in advanced nasopharyngeal carcinoma

**DOI:** 10.1038/s41419-023-06028-z

**Published:** 2023-08-10

**Authors:** Lizhen Liu, Peng Deng, Sailan Liu, Jing Han Hong, Rong Xiao, Peiyong Guan, Yali Wang, Peili Wang, Jiuping Gao, Jinghong Chen, Yichen Sun, Jianfeng Chen, Hai-Qiang Mai, Jing Tan

**Affiliations:** 1 Medical Research Institute, Guangdong Provincial People’s Hospital (Guangdong Academy of Medical Sciences), Southern Medical University, Guangzhou, China; 2 State Key Laboratory of Oncology in South China, Collaborative Innovation Center of Cancer Medicine, Guangdong Key Laboratory of Nasopharyngeal Carcinoma Diagnosis and Therapy, Sun Yat-sen University Cancer Center, Guangzhou, China; 3 Cancer and Stem Cell Biology, Duke-NUS Medical School, Singapore, Republic of Singapore; 4 Genome Institute of Singapore, A*STAR, Singapore, Republic of Singapore; 5 Laboratory of Cancer Epigenome, Division of Medical Sciences, National Cancer Centre Singapore, Singapore, Republic of Singapore

**Keywords:** Head and neck cancer, Cancer

## Abstract

Acquired resistance to chemotherapy is one of the major causes of mortality in advanced nasopharyngeal carcinoma (NPC). However, effective strategies are limited and the underlying molecular mechanisms remain elusive. In this study, through transcriptomic profiling analysis of 23 tumor tissues, we found that NOTCH3 was aberrantly highly expressed in chemoresistance NPC patients, with NOTCH3 overexpression being positively associated with poor clinical outcome. Mechanistically, using an established NPC cellular model, we demonstrated that enhancer remodeling driven aberrant hyperactivation of NOTCH3 in chemoresistance NPC. We further showed that *NOTCH3* upregulates *SLUG t*o induce chemo-resistance of NPC cells and higher expression of SLUG have poorer prognosis. Genetic or pharmacological perturbation of NOTCH3 conferred chemosensitivity of NPC in vitro and overexpression of NOTCH3 enhanced chemoresistance of NPC in vivo. Together, these data indicated that genome-wide enhancer reprogramming activates NOTCH3 to confer chemoresistance of NPC, suggesting that targeting NOTCH3 may provide a potential therapeutic strategy to effectively treat advanced chemoresistant NPC.

## Introduction

Nasopharyngeal carcinoma (NPC) is a malignancy of epithelia origin with widespread prevalence in specific geographical distribution. It occurs in high frequencies in Southeast Asian countries and Southern China. Multiple factors contribute to its pathogenesis, including EBV infection, genetic predisposition and environmental factors [1, 2]. Early-stage NPC is highly sensitive to radiotherapy and most of patients are curable. However, more than 70% newly diagnosed NPC are classified as advanced disease. Concurrent chemoradiotherapy is now the standard treatment for advanced stage NPC patients. Although high responsiveness is achieved initially, patients frequently develop acquired resistance to chemotherapy, resulting in local recurrence and distant metastasis, leading to high mortality [3–5]. Therefore, deciphering the resistance mechanisms of chemotherapy and exploring novel therapeutic strategies to overcome chemoresistance are imperative in the clinic.

Aside from genetic alterations, epigenetic changes also play a significant role in tumorigenesis and chemoresistance [6–8]. Epigenetic alterations include reversible modifications of DNA and histone proteins, such as DNA hypo/hypermethylation, histone modification, which can cause chromatin remodeling and altered gene expression. Genome-wide studies by the Cancer Genome Atlas project showed that NPC is more hypermethylated than 11 other cancer types [9], and hypermethylation at the promoter region of tumor suppressors is frequently reported in NPC [10–12]. However, other types of epigenetic dysregulation, other than DNA methylations, which may lead to chemoresistance of NPC remain elusive. Enhancer, marked by acetylation of the lysine residue at position 27 of H3 histone protein, is a short region of DNA which can be bound by transcription factors (TFs) to increase the transcription of an associated gene from far distance along the DNA. Epigenetic regulation of gene by enhancers is important for maintain cell identity. Dysregulation of enhancer has been found in many cancers, such as prostate cancer, lung cancer and leukemia [13–15] . One study has applied chromatin immunoprecipitation sequencing (ChIP-seq) analysis of NPC tissues and found that super-enhancer ETV6 correlated with prognosis of NPC patients [16]. However, how the enhancer landscape evolves and regulates gene expression during chemo-resistance of NPC has yet to be determined.

One of the important cellular pathways affected by epigenetic regulation is Notch signaling [17]. Notch signaling pathway is highly conserved through evolution and plays an important role in cell proliferation, apoptosis, differentiation and maintenance of somatic stem cells. Deregulation of Notch pathway has been demonstrated related to cancer initiation, relapse, metastasis and chemoresistance [18–20]. Notch has four receptors (NOTCH1, NOTCH2, NOTCH3 and NOTCH4) and five ligands (DLL1, DLL3, DLL4, JAG1 and JAG2) in mammals, which are differentially expressed and performing different functions in various tissues and tumors [21]. When the receptor of Notch binds to the ligand, it will be cleaved by γ-secretase and release the activated Notch intracellular domain (NICD) to the nucleus to activate downstream target genes. Among all four Notch receptors, NOTCH1 and NOTCH3 have been found highly expressed in NPC tissues compared with normal tissues [22, 23]. Moreover, inhibition of NOTCH3 increased the sensitivity of EBV related NPC cells to cisplatin. These points to the importance of the Notch receptors in NPC. However, the relevance and the regulation of NOTCH3 signaling in the chemoresistance of NPC have not been comprehensively investigated.

In this study, using patient samples and cellular models, we demonstrated that genome-wide enhancer reprogramming activates NOTCH3 signaling to confer chemo-resistance of NPC. Moreover, aberrant activation of NOTCH3-SLUG axis is correlated with chemo-resistance in NPC and poor prognosis of NPC patients, while targeting NOTCH3 signaling significantly resensitized NPC cells to paclitaxel. These data provide a potential therapeutic strategy to treat chemoresistant NPC patients.

## Results

### NOTCH3 is aberrantly highly expressed in chemoresistance NPC patients and associates with poor clinical outcome

**Fig. 1:**
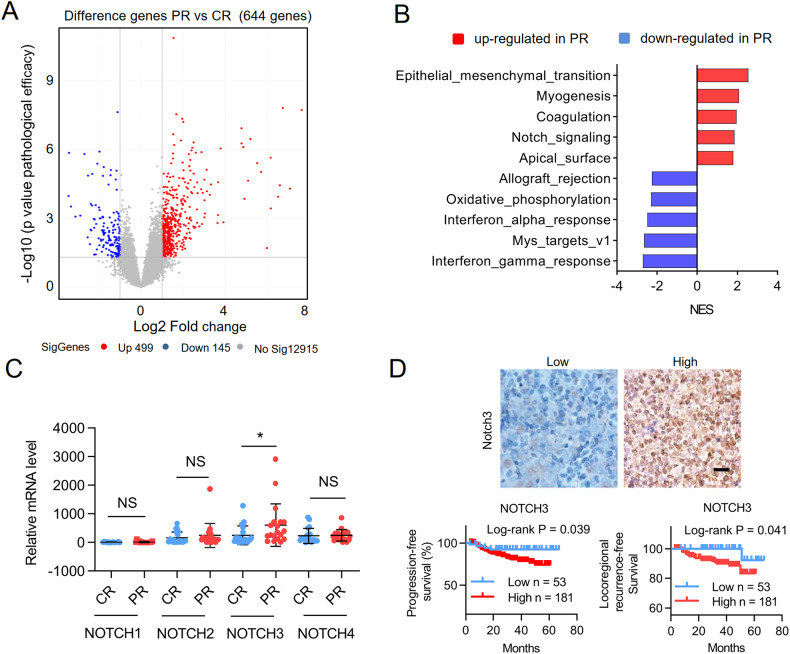
NOTCH3 is aberrantly highly expressed in chemoresistance NPC patients and associates with poor clinical outcome. **A** Volcano Plot of significantly altered genes from the RNA sequencing data of 22 late stage nasopharyngeal carcinoma patients, with 8 patients completely response (CR) to chemotherapy and 14 patients partially response (PR) to chemotherapy. **B** Pathway enrichment analysis of genes upregulated or downregulated in PR patients compared to CR patients. **C** qRT-PCR results of all four Notch receptors in patient samples. Bars represent the means ± SD, *n* = 3. *P < 0.05. **D** Up, representative IHC images of NOTCH3 in 234 nasopharyngeal carcinoma patients treated with chemotherapy. Scale bars, 30 μm. Down, Kaplan–Meier curves showing the impact of NOTCH3 expression on the progression-free survival and locoregional recurrence-free survival of 234 NPC patients. IHC staining of NOTCH3 was reviewed and scored by two independent pathologists blindly without knowing the clinical characteristics. IHC scores were calculated by multiplying the scores for the proportion of positively-stained tumor cells (1, <10%; 2, 10%–50%; 3, 50%–80%; 4, >80%) and staining intensity (0, no staining; 1, weak; 2, moderate; 3, strong) by each investigator, then averaged. The cutoff used in NOTCH3 survival analysis was selected by receiver operator characteristic curve (ROC) analysis. A score <2.5 was used to classify tumors with low NOTCH3 expression.

To investigate genes related to chemoresistance of advanced nasopharyngeal carcinoma, transcriptome analysis was performed on a total of 22 patients (Supplementary Table S4) treated with chemotherapy, of which 8 patients showed complete response (CR) and 14 patients showed partial response (PR). Totally 644 differentially expressed genes were found in PR versus CR (|log2 fold change| ≥ 1, adjusted *p*-value < 0.05) (Fig. 1A). Gene Set Enrichment Analysis (GESA) analysis revealed enriched Notch signaling pathway gene clusters in PR patients (Fig. 1B). The expression levels of all four Notch receptors in 40 NPC patient samples (Supplementary Table S5) were then determined by quantitative real time PCR (qRT-PCR) and only NOTCH3 was found significantly upregulated in PR patients compared with CR (Fig. 1C). Finally, the clinical relevance of NOTCH3 was assessed using immunohistochemistry (IHC) staining of NOTCH3 in 234 NPC patient samples with chemotherapy (Supplementary Table S6-7). Consistent with the reported localization of NOTCH3 [24], NOTCH3 protein was detected on the surface and inside of the cells (Fig. 1D). The Kaplan–Meier analysis results showed that high expression of NOTCH3 was negatively correlated with progression-free survival and locoregional recurrence-free survival of the patients (Fig. 1D). Collectively, these results suggest that aberrant activation of NOTCH3 might promote chemoresistance and malignancy of NPC.

### NOTCH3 overexpression promotes chemo-resistance of NPC cells

**Fig. 2:**
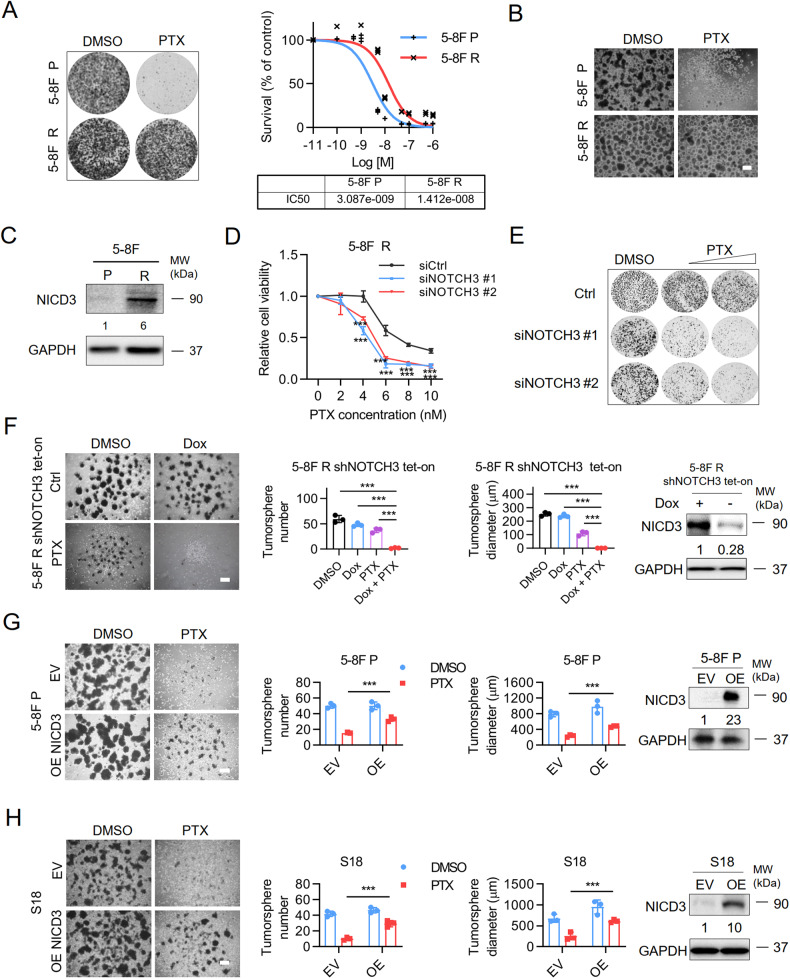
NOTCH3 overexpression promotes chemo-resistance of NPC cells. **A** Left, 2D colony formation assay of paired 5–8 F P and 5–8 F R cells under paclitaxel (PTX) treatment or not for 8 days. PTX concentration used was 2 nM. All the following experiments used the same concentrations of PTX, unless otherwise notified. Right, IC50 assay of 5–8 F P and 5–8 F R cells under different concentration of PTX treatment for 96 h. Cell viability was tested by CellTiter Glo reagent. **B** Tumorsphere assay of 5–8 F P and 5–8 F R cells with PTX treatment or without for 10 days. **C** Immunoblotting analysis of NICD3 expression in paired 5–8 F P and 5–8 F R cells. Results shown are representative image from three experiments. **D** Chemosensitivity to PTX by proliferation assay of 5–8 F R cells transfected with two different NOTCH3 siRNAs. Cell viability was determined by CellTiter Glo reagent after 96 h treatment. Bars represent the means ± SD (*n* = 3). *P < 0.05; **P < 0.01; ***P < 0.001. **E** Colony formation assay in 5–8 F R cells with two different NOTCH3 siRNAs treatment. DMSO treated cells were served as control. **F** Tumorsphere formation assay of shNOTCH3 tet-on 5–8 F R cells treated with doxycycline (Dox) in the presence or absence of PTX. Representative images (Left) and quantifications (Right). Bars represent the means ± SD (*n* = 3). Scar bars, 400 μm. *P < 0.05; **P < 0.01; ***P < 0.001. **G** Tumorsphere formation assay of 5–8 F P cells with NICD3 overexpression with or without PTX treatment. Representative images (Left) and quantifications (Right). Bars represent the means ± SD (*n* = 3). Scar bars, 400 μm. *P < 0.05; **P < 0.01; ***P < 0.001. **H** Tumorsphere formation assay of S18 cells and S18 with NICD3 overexpression cells treated with PTX or not. Representative images (Left) and quantifications (Right). Bars represent the means ± SD (*n* = 3). Scale bars, 400 μm. *P < 0.05; **P < 0.01; ***P < 0.001.

To determine the potential role of NOTCH3 in chemo-resistance of NPC, we established a paclitaxel-resistant (R) cell model through long time culture of 5–8 F parental (5–8 F P) cells in the presence of paclitaxel (PTX). The morphology and sensitivity to PTX of the resistant cells were confirmed (Fig. 2A-B). Intriguingly, we observed that 5–8 F R cells also became resistance to other NPC chemo-drugs, such as carboplatin and cisplatin (Supplementary Fig. S1A), which are commonly used in concurrent chemo-therapy of late stage NPC patients [25, 26]. Among all four Notch receptors, NOTCH3 was identified as the most highly expressed and upregulated receptor in 5–8 F R cells compared with 5–8 F P cells (Supplementary Fig. S1B). We then assessed Notch intracellular domain 3 (NICD3) expression in paired 5–8 F P and 5–8 F R cells. The accumulation of NICD3, which is released into the cytosol when the NOTCH3 receptor is activated, was observed in 5–8 F R cells, but not in 5–8 F P cells (Fig. 2C), which implied that NOTCH3 was aberrantly activated in 5–8 F R cells. Depletion of NOTCH3 with siRNAs conferred the chemo-sensitivity of 5–8 F R cells to paclitaxel in proliferation assay (Fig. 2D) and colony formation assay (Fig. 2E). Moreover, an inducible NOTCH3 knockdown system was established in 5–8 F R cells and conditional knockdown of NOTCH3 by doxycycline (Dox) significantly inhibited tumorsphere formation of the cells upon PTX treatment (Fig. 2F), while overexpression of NICD3 in 5–8 F P cells or another chemosensitive NPC cell line S18 cells remarkably promoted the tumorsphere formation capability in the presence of PTX (Fig. 2G, H). Together, these results indicated that NOTCH3 upregulation contributes to paclitaxel resistance in NPC.

### Enhancer reprogramming driven overexpression of NOTCH3 in chemo-resistant NPC cells

**Fig. 3:**
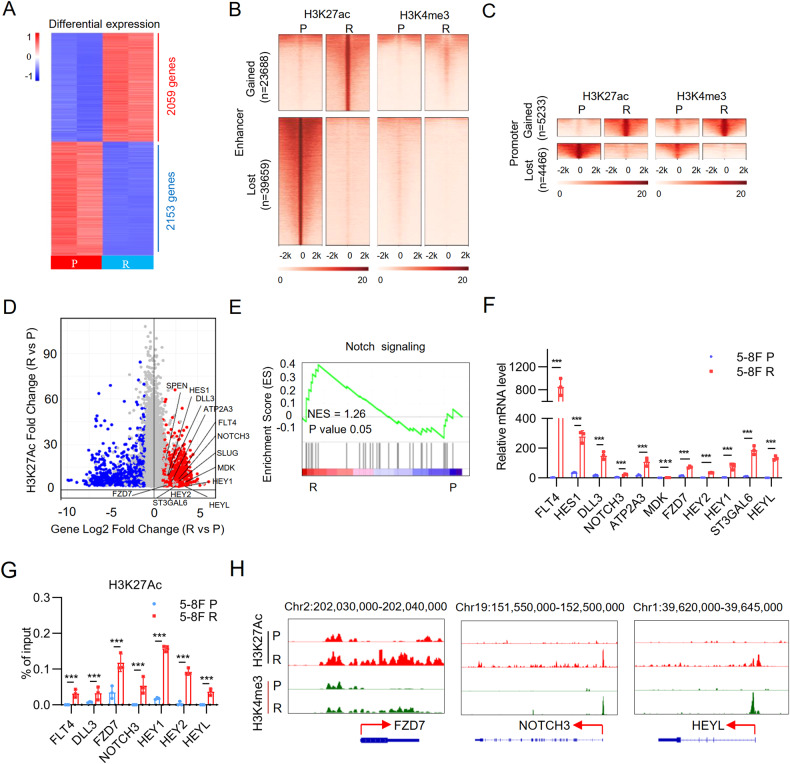
Enhancer reprogramming driven overexpression of NOTCH3 in chemo-resistant NPC cells. **A** Heatmap illustrating differentially expressed genes between 5–8 F P and 5–8 F R cells ( | log2 fold change | ≥ 1, adjusted *p*-value < 0.05). **B**, **C** Heatmaps of H3K27ac and H3K4me3 signals in 5–8 F P and 5–8 F R cells. The regions in which the H3K27ac and H3K4me3 levels gained or lost in 5–8 F R cells were identified in enhancer or promoter. Density heatmaps within ± 2.0 kb around the peak center were shown. **D** Integrative analysis of H3K27ac mark intensity and changes of corresponding gene expressions. Examples of genes related to Notch pathway have been labeled. **E** Gene set enrichment analysis (GSEA) shows enrichment of Notch signaling pathway in 5–8 F R cells. Normalized enrichment score (NES) and nominal *P*-value of GSEA are indicated. **F** qRT-PCR results of Notch pathway related genes in 5–8 F P and 5–8 F R cells. Bars represent the means ± SD, *n* = 3. *P < 0.05; **P < 0.01; ***P < 0.001.**G** Chip-qPCR analysis of H3K27ac bindings of Notch pathway related genes in 5–8 F P and 5–8 F R cells. Bars represent the means ± SD, *n* = 3. *P < 0.05; **P < 0.01; ***P < 0.001. **H** H3K27ac and H3K4me3 ChIP-seq peaks at FZD7, NOTCH3 and HEYL loci in 5–8 F P and 5–8 F R cells.

We then probed the mechanism underlying the aberrant upregulation of NOTCH3 in chemoresistance NPC. Transcription profiling of paired 5–8 F P and 5–8 F R cells identified 4211 differently expressed genes, with 2059 genes upregulated and 2153 genes downregulated in 5–8 F R cells compared with 5–8 F P cells ( | log2 fold change | ≥ 1, adjusted *p*-value < 0.05) (Fig. 3A). To determine whether the transcriptome changes in chemoresistance were caused by altered epigenetic landscape, we performed H3K27ac (marker of open chromatin regions) and H3K4me3 (marker of promoters) ChIP-seq in the paired cells. Promoters are defined as regions with both H3K27ac and H3K4me3 peaks that are within 2.0 kb of known transcription start site (TSS) and enhancers as regions with H3K27ac peaks that are more than 2.0 kb away from any known TSS. Altogether, 23,688 gained and 39,659 lost enhancers were identified in 5–8 F R cells (Fig. 3B). Similarly, 5233 gained and 4,466 lost promoters were found in 5–8 F R cells (Fig. 3C). Our integrative analysis of transcriptional profiles and chromatin landscapes revealed that differential gene expression between 5–8 F P and 5–8 F R cells were well correlated with changes in the H3K27ac levels (Fig. 3D). Meanwhile, gene set enrichment analysis (GESA) results indicated that Notch signaling pathway was aberrantly activated in 5–8 F R cells (Fig. 3E). This observation was further verified by qRT-PCR (Fig. 3F). Consistent with this result, ChIP-qPCR showed that H3K27ac increased at upregulated genes of Notch pathway in 5–8 F R cells (Fig. 3G). Enrichment of ChIP-seq peaks at the enhancer of several picked genes in chemo-resistant 5–8 F R cells also confirmed higher transcriptional activities (Fig. 3H). The upregulation of NOTCH3 is of importance, due to its clinical relevance. Histone Acetyltransferase (HAT) inhibitors have been shown to decrease H3K27ac levels and disrupt enhancer-driven transcriptions [27–29]. To further strengthen our hypothesis, we treated 5–8 F R cells with HAT inhibitors. *NOTCH3* expression was significantly reduced and in a dosage dependent manner (Supplementary Fig. S2A). These results together suggest that enhancer reprogramming activates NOTCH3 signaling to confer chemoresistance of NPC.

### *NOTCH3* upregulates *SLUG* to induce chemo-resistance in NPC

**Fig. 4:**
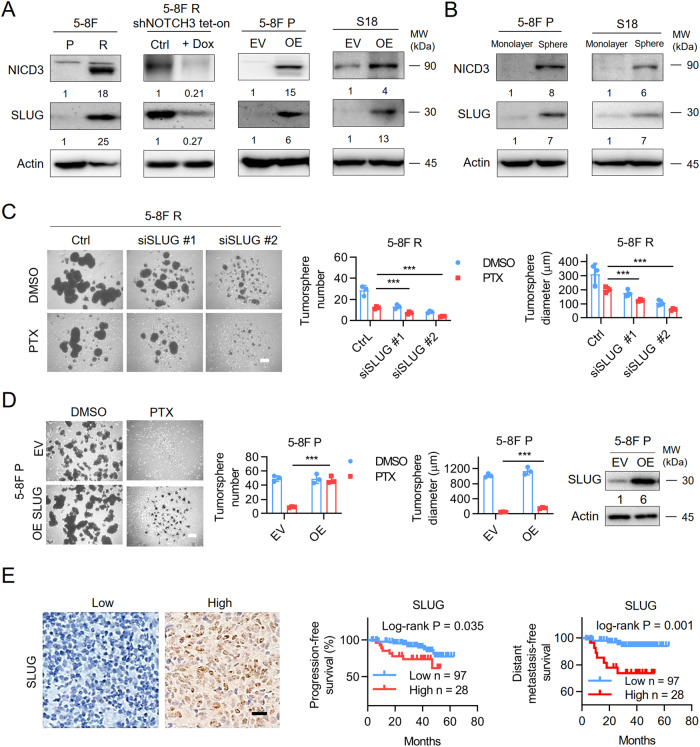
*NOTCH3* upregulates *SLUG* to induce chemo-resistance in NPC. **A** Immunoblotting analysis of NICD3, SLUG expression in different cells. Results shown are representative image from three experiments. Empty vector (EV), overexpression of NICD3 (OE). **B** Immunoblotting analysis of indicated protein expression in 5–8 F P and S18 cells in different culture conditions, monolayer or tumorsphere. Results shown are representative image from three experiments. **C** Tumorsphere formation assay of 5–8 F R cells treated with different SLUG siRNAs. Representative images (left) and quantification (right). Bars represent the means ± SD (*n* = 3). *P < 0.05; **P < 0.01; ***P < 0.001. Scale bars, 400 μm. **D** Tumorsphere formation assay of 5–8 F P cells with SLUG overexpression with or without PTX treatment. Representative images (Left) and quantifications (Right). Bars represent the means ± SD (*n* = 3). Scar bars, 400 μm. *P < 0.05; **P < 0.01; ***P < 0.001. **E** Left, representative IHC images of SLUG in 125 nasopharyngeal carcinoma patients treated with chemotherapy. Scale bars, 30 μm. Right, the Kaplan–Meier survival analysis for progression-free survival and metastasis-free survival and locoregional recurrence-free survival of 125 patients with nasopharyngeal carcinoma with high (red) or low (blue) SLUG expression levels. IHC staining of SLUG was blindly reviewed and scored by two independent pathologists without knowing the clinical characteristics. IHC scores were calculated by multiplying the scores for the proportion of positively-stained tumor cells (1, <10%; 2, 10%–50%; 3, 50%–80%; 4, >80%) and staining intensity (0, no staining; 1, weak; 2, moderate; 3, strong) by each investigator, then averaged. The cutoff used in SLUG survival analysis was selected by receiver operator characteristic curve (ROC) analysis. A score < 7 was used to classify tumors with low SLUG expression.

Previous studies reported that *SLUG* is a direct target of *NOTCH4* and the overexpression of SLUG has been shown to mediate chemoresistance in ovarian cells [30, 31]. Therefore, we hypothesized that *NOTCH3* might also upregulate *SLUG* to induce chemo-resistance in NPC. Compared with 5–8 F P cells, SLUG is expressed at higher levels in 5–8 F R cells. Knockdown of NOTCH3 in resistant cells reduced the expression of SLUG RNA and protein and while overexpression of NICD3 in parental cells resulted in significant induction of SLUG RNA and protein (Fig. 4A, Supplementary Fig. S3A-D). Interestingly, compared with monolayer culture, tumorsphere cultured S18 and 5–8 F P cells have higher expression of NICD3 and SLUG (Fig. 4B). Depletion of SLUG with siRNAs resulted in reduced tumorsphere formation of resistant cells under PTX treatment (Fig. 4C), while overexpression of SLUG significantly enhanced the tumorsphere formation ability of parental cells upon PTX treatment (Fig. 4D), suggesting that SLUG is important for maintaining the stemness of cancer cells during drug treatment. Moreover, the clinical relevance of SLUG in 115 nasopharyngeal carcinoma patient samples with chemotherapy (Supplementary Table S8-9) was assessed by IHC analysis in another cohort. The Kaplan–Meier survival analysis showed that high expression of SLUG is negatively correlated with the progression-free and metastasis-free survival of the patients (Fig. 4E). These results implied that activated NOTCH3-SLUG axis is significant for the chemoresistance of NPC.

### Chemical inhibitor targeting Notch signaling restores chemosensitivity of NPC cells in vitro

**Fig. 5:**
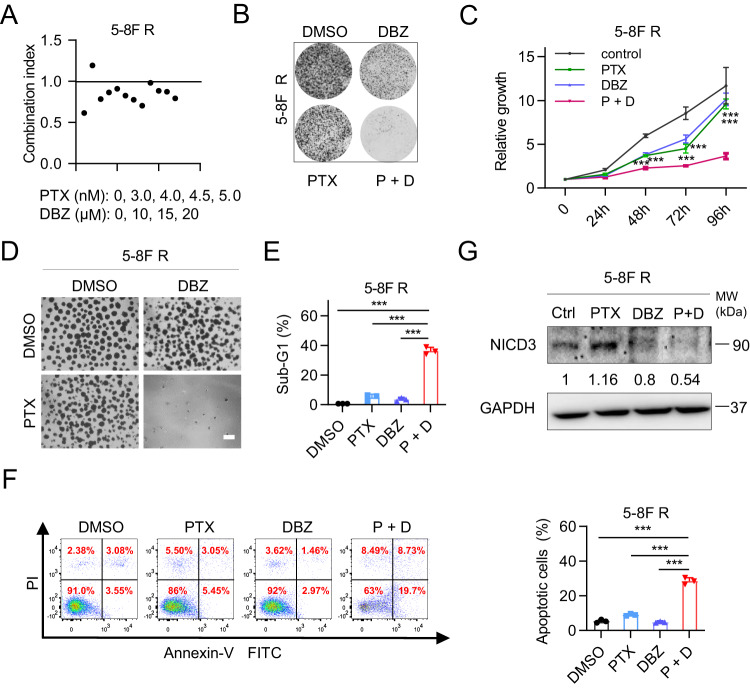
Chemical inhibitor targeting Notch signaling restores chemosensitivity of NPC cells in vitro. **A** Combination index (CI) of Dibenzazepine (DBZ) and paclitaxel (PTX) in 5–8 F R cells. The synergistic effect is demonstrated by CI value < 1.0. CI values were calculated with CalcuSyn software. Cells treated with indicated concentration of DBZ or PTX, single or in combination for 96 h. Cell viability was determined by CellTiter Glo reagent. **B** Colony formation assay of 5–8 F R cells treated with 25 μM DBZ in the presence or absence of 3.0 nM PTX for 8 days. All the following experiments used the same concentrations of DBZ or PTX, unless otherwise notified. PTX + DBZ (P + D). **C** Cell relative proliferation curve of 5–8 F R cells with different treatments. Data are shown as means ± SD (*n* = 3). *P < 0.05; **P < 0.01; ***P < 0.001. **D** Tumorsphere formation assay of 5–8 F R cells treated with Notch signaling inhibitor in the presence or absence of PTX for 12 days. Scale bar, 400 μm. **E** Sub-G1 population analysis in 5–8 F R cells treated with Notch signaling inhibitor, PTX or both for 72 h. Data are shown as means ± SD (*n* = 3). *P < 0.05; **P < 0.01; ***P < 0.001. **F** Annexin-V-PI staining to assess the apoptotic ratios of 5–8 F R cells under single or combined treatment for 72 h. Left, representative images of FACS analysis; Right, percentages of apoptotic cells in treatment groups as observed with flow cytometry. Bars represent the means ± SD, *n* = 3. *P < 0.05; **P < 0.01; ***P < 0.001. **G** Immunoblotting analysis of NICD3 under different treatments. Cells were collected after 72 h treatment. Results shown are representative image from three experiments.

To evaluate whether targeting NOTCH3 restores chemosensitivity of resistant NPC cells, we analyzed the chemo-sensitizing properties of Notch signaling inhibitor in paclitaxel resistant NPC cell line. Unfortunately, no specific NOTCH3 inhibitor has been developed currently. Drug combination index showed that the combination of Dibenzazepine (DBZ) and paclitaxel was synergistic (Fig. 5A). Colony formation assay and time-course proliferation assay indicated a dramatic increase in PTX sensitivity in 5–8 F R cells with combined treatment (Fig. 5B–C). In addition, the tumorsphere formation ability of 5–8 F R cell was effectively blocked by the combination treatment, indicating remarkable restoration of chemosensitivity (Fig. 5D). The combination treatment also remarkably increased the apoptosis of 5–8 F R cells (Fig. 5E, F). Moreover, decreased expression of NICD3 were observed under DBZ and combined treatment by western results (Fig. 5G). Similar results were obtained in other NPC cell lines (Supplementary Fig. S4A–D). These data demonstrated that Notch signaling inhibitor restored the sensitivity to PTX in 5–8 F R cells, inhibited cell proliferation and promoted cell death.

### NOTCH3 overexpression enhances chemoresistance of NPC in vivo

**Fig. 6:**
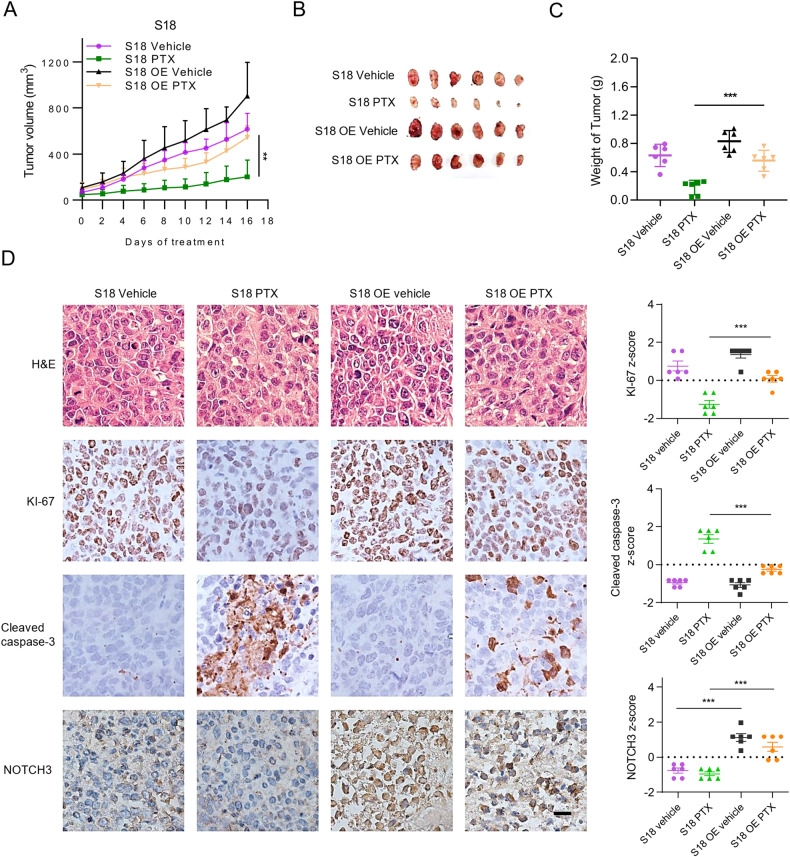
NOTCH3 overexpression enhances chemoresistance of NPC in vivo. **A** Xenograft tumor growth curve of S18 cells and S18 with stable overexpression of NICD3 (S18 OE) cells under different treatments. S18 and S18 OE cells were treated with 5 mg/kg paclitaxel or without for 16 days, as descried in Material and Methods. Error bars, mean ± SEM (*n* = 6 per group). *P < 0.05; **P < 0.01 (independent *t*-test). **B** Tumors were presented by photographs. Each group consists of 6 mice. **C** Bar graph shows the tumor weight at the 16th day post-treatment. Data are presented as the mean ± SD. *P < 0.05; **P < 0.01; ***P < 0.001 (independent *t*-test). **D** HE, KI-67, cleaved caspase-3 and NOTCH3 IHC staining of xenograft tumor. Up: Representative images. Scale bars 20 μm. Down: Z-score of KI-67, cleaved caspase-3, NOTCH3 in tumor sections with different treatments. IHC scores were calculated by multiply the scores for the proportion of positively-stained tumor cells (1, <10%; 2, 10%–50%; 3, 50%–80%; 4, >80%) and staining intensity (0, no staining; 1, weak; 2, moderate; 3, strong) by each investigator, then averaged. Z-score of IHC was defined by using the formula *z* = (x − μ)/ σ, where x is the raw IHC score, μ is the population mean, and σ is the population SD. Two-tailed *t-*test was used to calculate *p*-values. *P < 0.05; **P < 0.01; ***P < 0.001.

Due to that 5–8 F R cells had a very low tumorigenesis rate, to further verify the role of NOTCH3 in chemoresistance in vivo, S18 cells and S18 with stable overexpression of NICD3 (S18 OE) cells were engrafted subcutaneously to nude mice. Mice with S18 and S18 OE xenograft tumors were treated with or without paclitaxel. Notably, PTX treatment reduced the tumor sizes of S18 cells significantly but not S18 OE cells, indicating that enhanced NOTCH3 activation and signaling confers chemoresistance of NPC in vivo (Fig. 6A–C, Supplementary Fig. S5A–B). IHC results demonstrated that cell proliferation (represented by KI-67 expression) was remarkably reduced and apoptosis (represented by cleaved caspase-3 expression) was increased in S18 xenograft tumors treated with PTX compared with S18 OE xenograft (Fig. 6D). Moreover, the overexpression of NOTCH3 in S18 OE groups were confirmed by IHC (Fig. 6D). These data certified that NOTCH3 overexpression plays an important role in the chemoresistance of NPC in vivo.

## Discussion

The incidence of advanced NPC is rising in Southeast Asian countries and in Southern China, yet therapeutic strategies remain limited and ineffective, resulting in high mortality. Although the combined treatment of chemo-radiotherapy produces a satisfying survival rate initially, metastasis and chemo-resistance inevitable develops. In the present study, we seek to unravel the molecular mechanism of chemoresistance in NPC, which may provide potential therapeutic targets to improve clinical outcomes. Through analysis of NPC tumor tissues and cellular models, we reported that enhancer remodeling activates NOTCH3 to confer chemo-resistance in NPC. Moreover, genetic or pharmacological perturbation of NOTCH3 could potentiate the sensitivity to paclitaxel in NPC. Thus, we propose that targeting NOTCH3 signaling will help overcome chemoresistance in NPC.

The current work focused on advanced chemo-resistant NPC and epigenetic regulation. To our knowledge, this is the first report demonstrated that enhancer remodeling driven aberrant activation of NOTCH3 to enhance the expression of SLUG, which confers chemoresistance of NPC. Epigenetic signature changes are dynamic and reversible, making chemoresistance-specific enhancers ideal therapeutic targets since they wouldn’t affect normal tissues. At therapeutic perspective, NOTCH3 enhancer could be a best potential target in advanced NPC. Gamma secretase inhibitor (GSI) gained attention in cancer treatment during the past decade [32–34]. It acts by inhibiting the cleavage of γ-secretase, which results in blocking Notch signaling pathway. DBZ is a type of GSI, our data showed that DBZ treatment reduced the expression level of NICD3 in 5–8 F R cells and sensitized resistance cells to PTX. Unfortunately, it is not a specific inhibitor for NOTCH3 and the usage of GSI is limited due to its toxicity [35], better inhibitor should be developed in the future.

SLUG is a member of SNAIL family of zinc-finger transcription factors and its deregulation has been found in several types of human cancer [36–38]. In addition to regulating epithelia-mesenchymal transition, researchers have shown that SLUG also plays a role in maintenance of the stemness properties of tumor cells. The stem-like phenotype of cancer cells has been proven related with resistance to chemotherapy and radiotherapy in various cancers [39–41]. Our study showed that *SLUG* is a downstream target of *NOTCH3* with clinical relevance in NPC. Moreover, SLUG overexpression was correlated with the increased tumorsphere formation ability of NPC cells upon PTX treatment, which suggested that SLUG maintained the stemness of NPC cells in the presence of PTX. Similar with our results, SLUG upregulation has been implicated in radiotherapy resistance in NPC previously [42–44]. Taken together, our data firstly showed that NOTCH3-SLUG axis is correlated with chemo-resistance in NPC and poor prognosis of NPC patients.

The present study also has limitations, including the inability to find another NPC cell line with NOTCH3 overexpression. Actually NPC is consistently associated with Epstein-Barr virus, however, most if not all the reported NPC cell lines have lost their EBV episomes and became EBV-negative upon in vitro propagation. So it’s hard using in vitro models to mimic clinical situation.

**Fig. 7:**
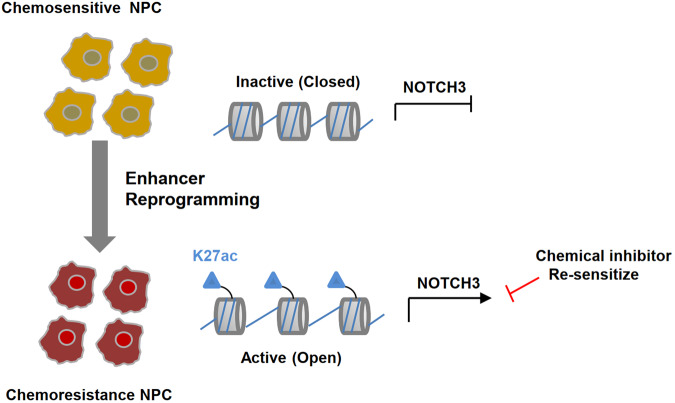
A schematic summary of the findings of this work. Genome-wide enhancer reprogramming activates NOTCH3 signaling to confer chemoresistance of NPC, suggesting that targeting NOTCH3 could provide a potential therapeutic strategy to effectively treat chemoresistant NPC.

In summary, our study demonstrated that genome-wide enhancer reprogramming activates NOTCH3 signaling to confer chemo-resistance of NPC (Fig. 7). The overexpression of NOTCH3 and SLUG were correlated with chemoresistance of NPC and poorer prognosis of NPC patients. Importantly, genetic depletion or pharmacological inhibition of NOTCH3 restores the chemosensitivity of NPC, which may provide a potential therapeutic strategy for advanced NPC patients.

## Materials and methods

### Patient samples, cell culture, and reagent

This study was approved by the Institutional Ethical Review Board of Guangdong Provincial People’s Hospital (KYZ202157201). Written informed consents were obtained from all patients. Human nasopharyngeal carcinoma cell lines 5–8 F, 6–10B, CNE2, S26, S18 and HEK293T cells were grown in RPMI1640 (Invitrogen) or DMEM (Invitrogen) supplemented with 10% FBS and 1% penicillin–streptomycin (Gibco BRL). Cells were maintained at 37 °C in a 5% CO_2_ incubator and tested for mycoplasma contamination every month. All the cell lines used in this study had been authenticated and were generously provided by Dr. M. Zeng (Sun Yat-sen Univeristy Cancer Centre). To establish paclitaxel-resistant cell lines, 5–8 F cells were treated with paclitaxel for 3-months by escalating concentration, until certain target concentration was achieved. Resistant cells were maintained under 2.5 nM paclitaxel exposure until experiments were performed. Dibenzazepine (DBZ), SGC-CBP30 and dCBP1 were purchased from TargetMol.

### RNA sequencing (RNA-seq) and data analysis

Total RNA was extracted from NPC patient samples or 5–8 F P and 5–8 F R cells by Trizol (Invitrogen), followed by purification using RNeasy Mini Kit (Qiagen). RNA-seq library were constructed using Truseq Stranded Total RNA Gold library kit (Illumina) according to manufacturer’s protocol. The libraries were sequenced using an Illumina HiSeq2500 platform with paired-end reads of 150 bases. For data analysis, clean reads gained with fastp software (version 0.12.5) [45] were aligned to human reference genome (GRCh38, hg38 with STAR (version: 2.7.0 f)) [46]. RSEM (version: 1.3.3) was used to calculate the transcripts value for each gene [47]. Differentially expressed genes were called by DESeq2 R package (version:1.28.1) with | log_2_ fold change | ≥ 1 and adjusted *p*-value < 0.05. Gene Set Enrichment Analysis was assessed by a GSEA program [48].

### Quantitative real-time PCR (qRT-PCR)

For qRT-PCR, total RNA was extracted as described above and reverse-transcribed into cDNA using TransScript All-in-one First-Stand cDNA Synthesis SuperMix for qPCR kit (Transgene Biotech). Quantitative real-time PCR was performed with SYBR Green(Vazyme) on a Bio-Rad PCR Detection System. 18 S was used as internal control. All experiments were performed in biological triplicates unless stated otherwise. Sequences of qRT-PCR primers are listed in Supplementary Table S1.

### Chromatin immunoprecipitation DNA-sequencing (ChIP-seq) and data analysis

5–8 F P and 5–8 F R cells were seeded into T75 flask for 72 h before collected for ChIP-sequencing. 1 × 10^6^ cells were used for each ChIP-sequencing experiment and the cells were crosslinked for 10 min with 1% formaldehyde at room temperature. The cells were then lysed with 1% SDS lysis buffer (50 mM Tris-HCl pH 8.0, 10 mM EDTA, 1% SDS) and sonicated on ice using Bioruptor (Diagenode) for 16 cycles (on and off session at 30 s each). Supernatant was collected and precleared with protein G Dynabeads (Invitrogen) for 2 h at 4 °C. Then the chromatin extract was incubated with specific antibody-binding magnetic beads overnight at 4 °C. The beads were washed with low and high salt buffer and chromatin was eluted from the beads with elution buffer (50 mM Tris-HCl pH 8.0, 10 mM EDTA, 1%SDS) after incubating for 40 min at 68 °C with agitation. Precleared chromatin without incubation with antibody-binding beads was served as input. Reversed cross-link was performed and DNA were isolated using Phenol-Chloroform-Isoamyl alcohol (Thermo Fisher Scientific) and ethanol precipitation. ChIP-DNA obtained were used for ChIP-qPCR and sequences of the primers used were shown in Supplementary Table S2.

Input and ChIP DNA were amplified using GenomePlex® Single Cell Whole Genome Amplification kit. After amplification, DNA was purified by PCR product purification kit (Qiagen) and the concentration of amplified DNA was determined by PicoGreen. After digestion by BpmI restriction enzyme, 30 ng DNA was used for library preparation using NEBNext® ChIP-seq Library Prep Master Mix Set for Illumina® kit. KAPA library Quantification and qPCR were performed for specific genes, as quality control. Libraries were sequenced using an Illumina HiSeq2500 platform with paired-end reads of 150 bases.

For ChIP-seq data analysis, reads were aligned to the human reference genome (GRCh38, hg 38) using Bowtie2 (version 2.3.2) [49]. MACS2 (version: 2.1.2) was employed to call narrow peaks of H3K27ac and H3K4me3 with default settings (-q 0.01) [50]. ChIPseeker (version 1.14.2) [51] was used to annotate the peaks and visualization of the data was performed by deepTools [52]. Promoters are defined as regions with both H3K27ac and H3K4me3 peaks that are within 2.0 kb of known transcription start site (TSS) and enhancers as regions with H3K27ac peaks that are >2.0 kb away from any known TSS. Alignments for specific gene were visualized using Integrative Genomics Viewer (version 2.4.19) [53].

### Colony formation, tumorsphere formation, IC50 and cell proliferation assay

For colony formation assay, 1 × 10^4^ cells were plated in 6-well plate 24 h. before the indicated reagents were added. When the control group reached 100% confluence, colonies were fixed with methanol and stained with Crystal Violet (Sigma) for colony visualization. For tumorsphere formation assay, cells were trypsinized and passed through 0.4 μm cell strainer to achieve single-cell suspension. Cells was cultured (1 × 10^4^ cells/well) in 6-well ultra-low-attachment plates (Corning) with MammoCult Medium (STEMCELL Technologies, Vancouver, BC) supplemented with heparin (1:500) and fresh hydrocortisone (0.5 μg/ml). Tumorspheres were cultured for 10–12 days and stained with 2-(4-iodo-phenyl)-3-(4-nitrophenyl)-5-phenyl-2H-tetrazolium chloride (INT) (Sigma-Aldrich) and photographed. Images were taken with Olympus microscope IX71 and colony quantification were performed using Olympus CellSens Dimension software. For IC50 assay, 2 × 10^3^ cells per well were seeded in 96-well plate 24 h before treatment with different dosages of PTX (0.01, 0.05, 1, 5, 10, 50, 100 and 500 nM). 96 h later, cells were lysed with CellTiter Glo and chemiluminescent signal was detected with a microplate reader. For proliferation assay, 2 × 10^3^ cells per well were seeded in 96-well plate. 24 h later, cells were treated with the indicated reagents. The number of viable cells were measured by CellTiter Glo reagent at the indicated time points.

### Combination index assay

Synergistic effects of combined drug treatment were assessed by combination index (CI) assay, where CI < 1.0 represents synergy. Briefly, 5–8 F R cells were exposed to indicated concentrations of Notch signaling inhibitor with or without paclitaxel for 96 h. CI values were determined by the inhibition rate of the cells calculated with CalcuSyn software.

### Immunoblotting analysis

Cells were lysed in RIPA buffer with protease inhibitors. Protein concentration was determined by a Bradford Assay kit (Bio-rad). Protein was separated by 8% or 12% SDS-PAGE and transferred onto a PVDF membrane. The membrane was blocked in 5% skim milk or 5% BSA, followed by primary antibody incubation overnight. The primary antibodies used were: NOTCH3 (CST, 5276), SLUG (CST, 9585), Actin (CST, 4359) and GAPDH (CST, 2118). The secondary antibodies used were horseradish peroxidase-conjugated anti-rabbit and anti-mouse (GE Healthcare Life Science, NA934 and NA931). The bands were detected using chemiluminescence with ChemiDox^TM^ MP Imaging System (Bio-Rad).

### Flow cytometric analysis

PI staining was done to quantify the sub-G1 population, which can reflect the extent of cell death. Cells were seeded in 6-well plates 24 h prior to treatment with different compounds. After 72 h, cells then collected, fixed and stained with PI (50 μg/mL). The stained cells were analyzed by SP6800 Spectral Cell Analyzer (Sony) and quantified by using the FlowJo software.

Cell death was also detected by flow cytometry using Annexin V-FITC/PI double staining (BD Biosciences) using the manufacturer’s protocol. The stained cells were immediately analyzed by flow cytometric analysis.

### siRNA, shRNA and plasmid transfection

Paclitaxel-resistant cells were transiently transfected with siRNAs targeting NOTCH3, SLUG or with negative control siRNA obtained from Sangon Biotech Company, using lipofectamine RNAimax reagents (Thermo Fisher Scientific). Transfection was performed according to the manufacturer’s instructions. The sequences of the siRNAs are shown Supplementary Table S3.

To generate NOTCH3 inducible knockdown cell lines, an shRNA from the TRIPZ Dox-inducing lentiviral shRNA system was used. The lentivirus vectors were co-transfected with two packaging vectors psPAX2 and pMD2.G at a ratio of 2 μg: 1 μg: 0.5 μg into packaging cell HEK293T. After 48 h, supernatant containing virus were collected and filtered. The filtered virus was used to infect the host cells. 48 h after infection, cells were selected by puromycin (1.0 μg/mL). To induce NOTCH3 knockdown, 1.0 μg/mL doxycycline was added. The sequence of shRNA targeting NOTCH3 is shown in Supplementary Table S3. The expression plasmid of NICD3 is a kind gift from Prof. Suling Liu (Fudan University Shanghai Cancer Center). Briefly, cDNA of NOTCH3 intracellular domain (amino acids 1663–2321) was synthesized at Sangon Biotech (Shanghai, China) and cloned into pLVX-puro vector. Similarly, the coding sequences for SLUG were amplified from cDNA obtained from HEK293T cells and then inserted into pLVX-puro plasmid. The empty vector (pLVX-puro) and expression plasmids were transfected with Lipofectamine 2000 (Thermo Fisher Scientific) reagents into 5–8 F R or S18 cells.

### Immunohistochemistry (IHC) analysis

The procedure of IHC was performed as previously described [54]. Briefly, the slides were incubated overnight at 4 °C with primary antibodies and followed by 60-minnute incubation with rabbit polymer detection system kit or mouse polymer detection system kit (ZsbioCommerce Store, PV-6001, PV-6002). IHC staining was reviewed and scored by two independent pathologists blindly without knowing the clinical characteristics. IHC scores were calculated by multiplying the scores for the proportion of positively-stained tumor cells (1, <10%; 2, 10%–50%; 3, 50%–80%; 4, >80%) and staining intensity (0, no staining; 1, weak; 2, moderate; 3, strong) by each investigator, then averaged. The cutoff used in NOTCH3 and SLUG survival analysis was selected by receiver operator characteristic curve (ROC) analysis. A score <2.5 was used to classify tumors with low NOTCH3 expression and a score <7 was used to classify tumors with low SLUG expression. Z-score of IHC was defined by using the formula z = (x-μ) / σ, where x is the raw IHC score, μ is the population mean, and σ is the population SD. The antibody used were KI-67 (Zsbio Commerce Stor, ZA-0502), cleaved-caspase 3 (CST, 9661), SLUG (Novus, NBP2-03886) and NOTCH3 (Abcam, ab23426).

### Animal studies

Female BALB/c nude mice (5–6 weeks) were purchased from Beijing Vital River Laboratory Animal Technology Company. All experiments were conducted in compliance with animal protocols approved by the Ethics of Animal Experiments of Guangdong Provincial People’s Hospital. Tumors were measured by Vernier caliper every two days. And tumor volume was calculated with the following formula: tumor volume (V) = width × width × length × 0.537. For S18 xenograft model, 2 × 10^6^ cells were subcutaneously injected into the left flank of the mice. After the tumors volume reached 100 mm^3^, the mice were randomly divided into two groups: vehicle-treated group (*n* = 6) and paclitaxel-treated (5 mg/kg) group (*n* = 6). For S18 cells with NICD3 overexpression (S18 OE), same cells number as S18 were implanted subcutaneously at the same time. And mice were divided randomly into two groups for drug treatment simultaneously: vehicle alone (*n* = 6), paclitaxel treatment alone (*n* = 6). Paclitaxel was given every two days by intraperitoneal injection. Mice were sacrificed by CO_2_ inhalation after the tumor volume of S18 control group reached 1000 mm^3^ and tumors were harvested for further analysis. No statistical method was used to predetermine the sample size for this experiment. No data was excluded and the investigator was blinded to the group allocation during the experiment.

### Statistical analysis

The two-tailed student’s *t*-test was used to calculate differences between experimental groups. *P* < 0.05 was considered statistically significant unless stated otherwise. All statistical analyses were done using GraphPad Prism version 7.0 and all in vitro experiments were repeated at least 3 times. Protein expression levels were quantified using Image J software, and the relative quantity of protein was calculated.

## Supplementary information

Supplementary Figures S1-S5

Supplementary Tables S1-S9

Western uncut

## Data Availability

ChIP-seq and RNA-seq data are deposited in GEO: GSE153125 and GSE21402.
