# Supplementary material for: Enhancer remodeling activates NOTCH3 signaling to confer chemoresistance in advanced nasopharyngeal carcinoma
Source: Cell Death Dis. 2023 Aug 10;14(8):513. doi: 10.1038/s41419-023-06028-z (PMC10415329; doi:10.1038/s41419-023-06028-z)
Supplement: Supplementary file 1 — Supplementary Figures S1-S5 [file 41419_2023_6028_MOESM1_ESM.pdf]

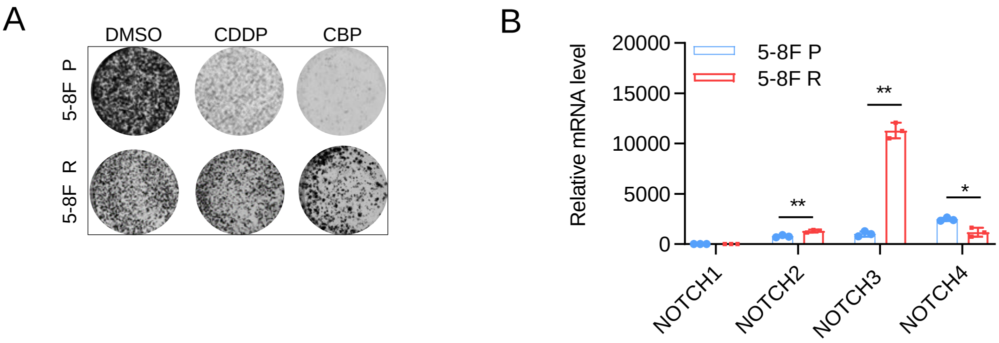

Supplementary Figure 1

5-8F R cells acquired resistance to other chemo-drugs used in NPC with NOTCH3 extremely highly upregulated. A, Colony formation assay of 5-8F P and 5-8F R under different chemo-drug treatments for 8 days. CDDP, cisplatin; CBP, carboplatin. **B**, qRT-PCR results of all four Notch receptors in 5-8F P and 5-8F R cells. Bars represent the means ± SD, n = 3. *, $P < 0.05$; **, $P < 0.01$.

A

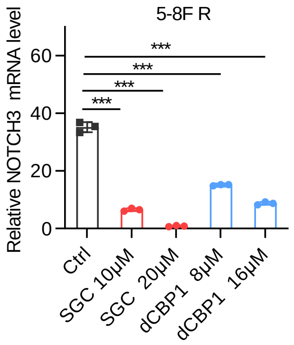

Supplementary Figure 2

Histone Acetyltransferase (HAT) inhibitors reduce the expression of NOTCH3 in 5-8F R cells. A, qRT-PCR results of *NOTCH3* in 5-8F R cells with different Histone Acetyltransferase (HAT) inhibitors treatments. Cells were collected after 72 hr. Bars represent the means ± SD, n = 3. *, $P < 0.05$; **, $P < 0.01$; ***, $P < 0.001$. SGC-CBP30 (SGC).

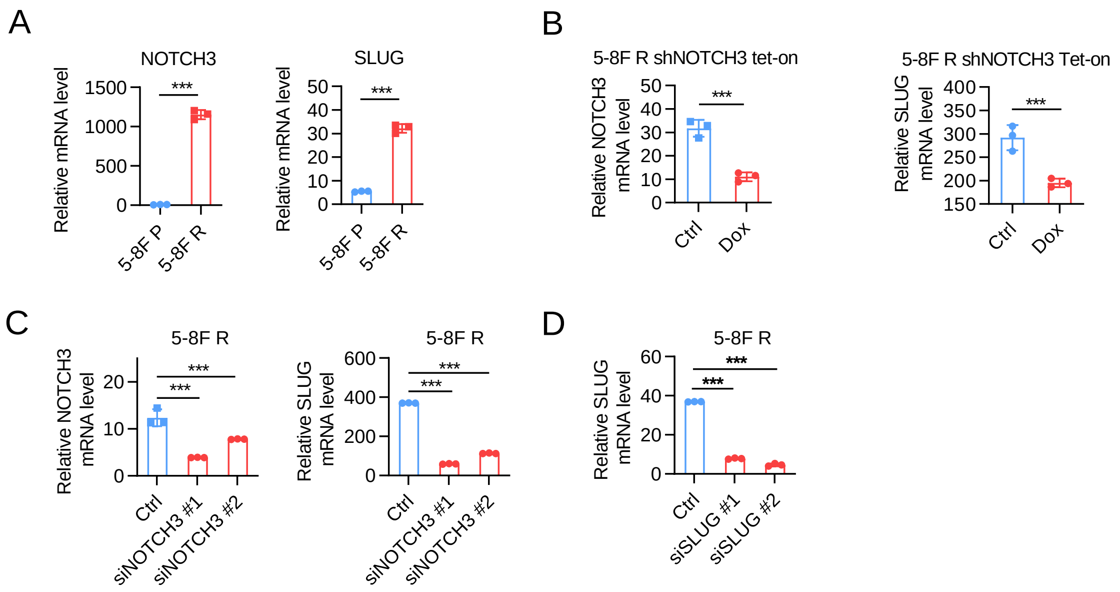

Supplementary Figure 3

***NOTCH3* regulates *SLUG* expression in NPC. A,** qRT-PCR analysis of *NOTCH3* and *SLUG* expression of 5-8F Parental (5-8F P) and 5-8F Resistant (5-8F R) cells. Bars represent the means ± SD, n = 3. *, $P < 0.05$; **, $P < 0.01$; ***, $P < 0.001$. **B,** qRT-PCR analysis of *NOTCH3* and *SLUG* expression of 5-8F R shNOTCH3 tet-on cells with or without Dox treatment. Bars represent the means ± SD, n = 3. *, $P < 0.05$; **, $P < 0.01$; ***, $P < 0.001$. **C,** qRT-PCR analysis of *NOTCH3* and *SLUG* expression of 5-8F R cells treated with different NOTCH3 siRNAs. Bars represent the means ± SD, n = 3. *, $P < 0.05$; **, $P < 0.01$; ***, $P < 0.001$. **D,** qRT-PCR analysis of *SLUG* expression of 5-8F R cells treated with different SLUG siRNAs. Bars represent the means ± SD, n = 3. *, $P < 0.05$; **, $P < 0.01$; ***, $P < 0.001$.

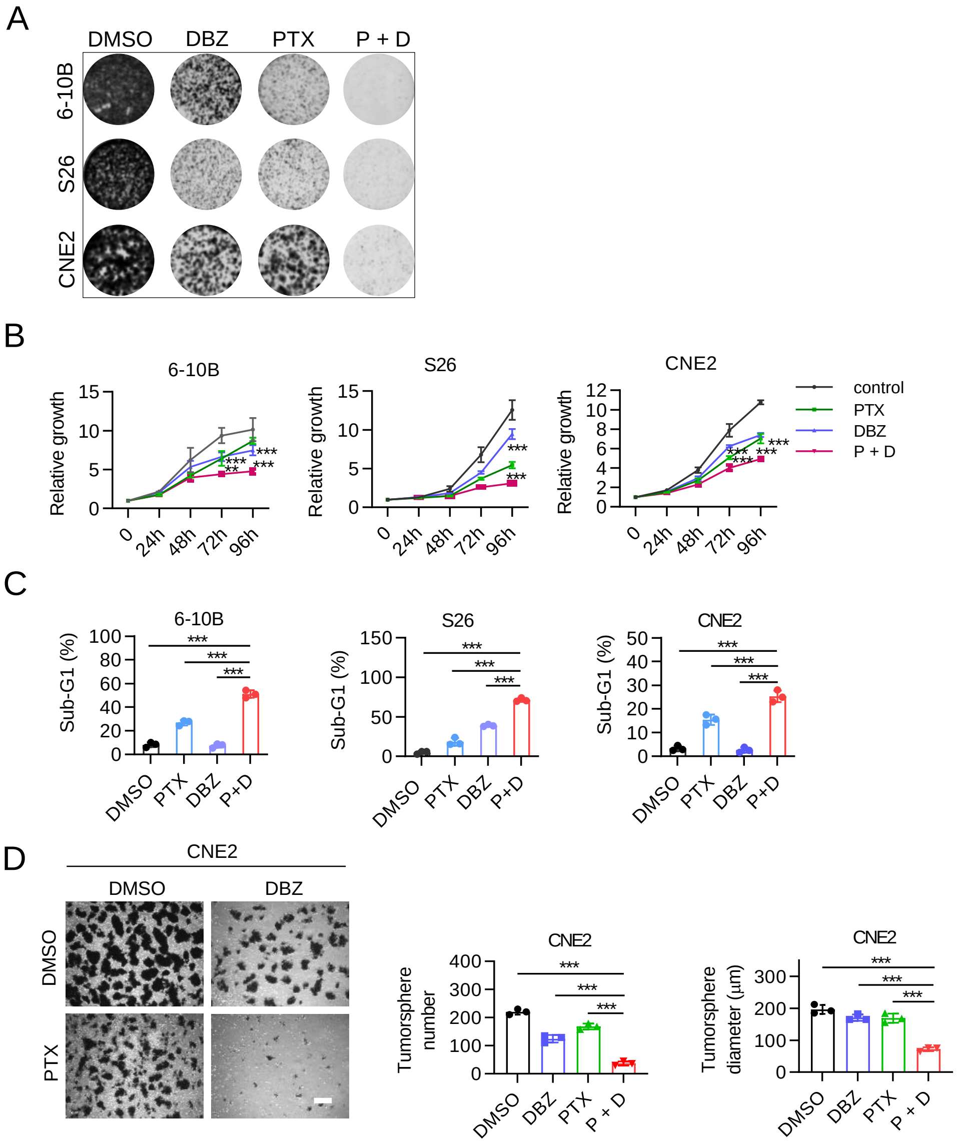

Supplementary Figure 4

Chemical inhibitor targeting Notch signaling restores chemosensitivity of NPC cells in vitro. A, Colony formation assay of NPC other cell lines under combined treatment. PTX concentration used were optimized for each cell. PTX + DBZ (P + D). **B,** The growth curves of different NPC cell lines under Notch signaling inhibitor treatment with or without PTX. The concentration of DBZ used were 25 μM. The paclitaxel (PTX) concentration used was 1.0 nM for 6-10B, S26 and CNE2 cells. The same concentration was used for the following experiments unless otherwise notified. Cell viability was determined using CellTiter Glo reagent. Data are shown as means ± SD (n = 3). *, $P < 0.05$; **, $P < 0.01$; ***, $P < 0.001$. **C,** Sub-G1 population analysis in different NPC cell lines treated with Notch signaling inhibitor, PTX or both for 72 hr. Data are shown as means ± SD (n = 3). *, $P < 0.05$; **, $P < 0.01$; ***, $P < 0.001$. **D,** Tumorsphere formation assay of CNE2 cells under Notch signaling inhibitor treatment with or without paclitaxel for 10 days. Representative images (Left) and quantifications (Right). Bars represent the means ± SD (n = 3). *, $P < 0.05$; **, $P < 0.01$; ***, $P < 0.001$. Scar bars, 400 μm.

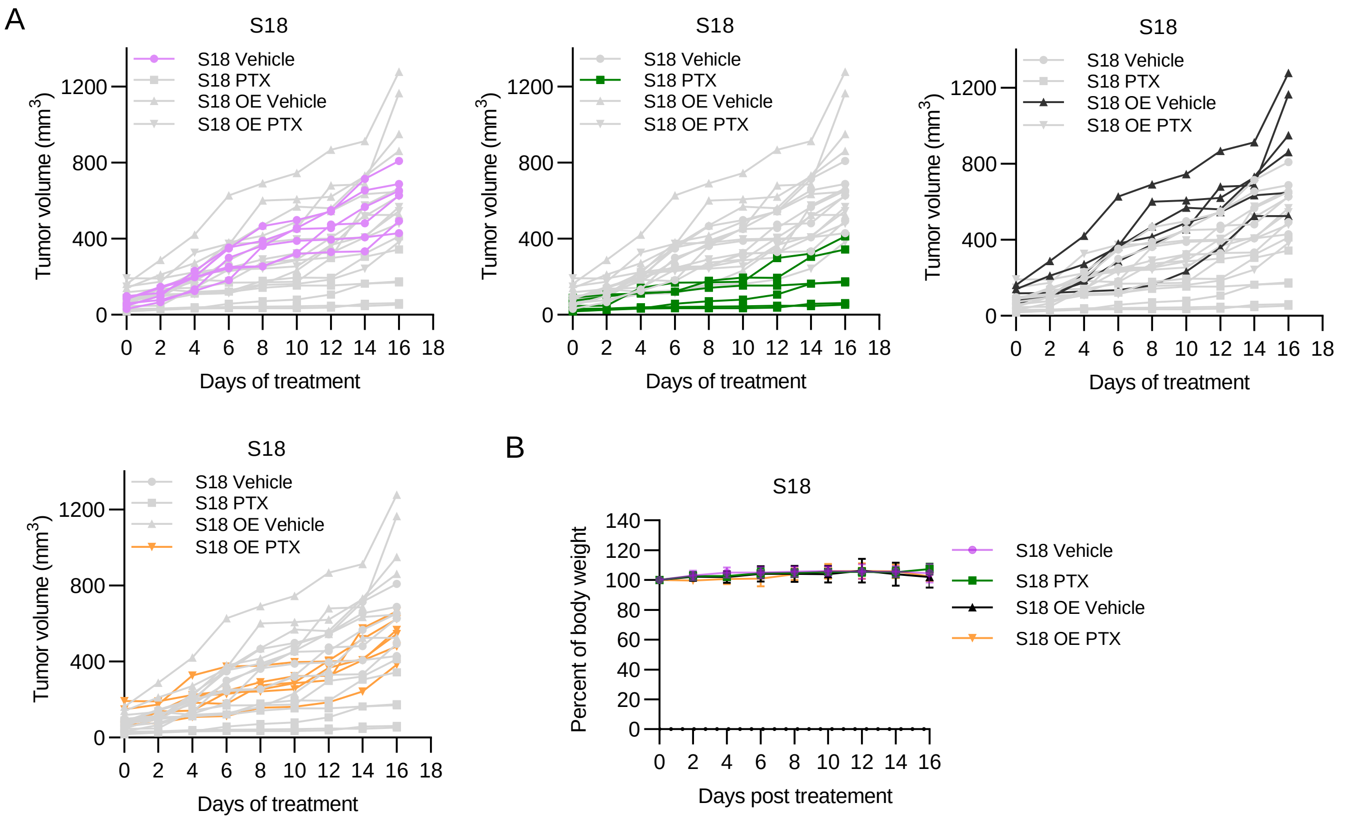

Supplementary Figure 5

NOTCH3 overexpression enhances chemoresistance of NPC in vivo. A, The growth curve of individual mice with different treatments. **B,** Percentage Change in body weight of the mice during the 16 days of treatment. Error bars represent mean ± SEM (n = 6 per group).
