# Supplementary material for: Enhancer remodeling activates NOTCH3 signaling to confer chemoresistance in advanced nasopharyngeal carcinoma
Source: Cell Death Dis. 2023 Aug 10;14(8):513. doi: 10.1038/s41419-023-06028-z (PMC10415329; doi:10.1038/s41419-023-06028-z)
Supplement: Supplementary file 2 — Supplementary Tables S1-S9 [file 41419_2023_6028_MOESM2_ESM.pdf]

Supplementary Table 1. The gene-specific primers used in qRT-PCR

**Gene**	**Forward Primer 5'-3'**	**Reverse Primer 5'-3'**
18s	CGAACGTCTGCCCTATCAACTT	ACCCGTGGTCACCATGGTA
FLT4	CAACCAGACAGACAGTGGGA	GCCGCTTTCTTGTCTATGCCT
HES1	CTGAGCACAGACCCAAGTGT	GAGTGCGCACCTCGGTATTA
DLL3	CCCTTCCTCGATTCTGTCCG	ACCTCCTCAAGCCCATAGGT
NOTCH3	CGTCAGTGTGAACTCCTCTCC	AGATACCATGAGGGCCACAG
ATP2A3	CGGAACCACATGCACGAAGA	ACCTCGGGCCTGTCATTTAT
MDK	GAGTCGCCTCTTAGCGGATG	GCCGCCCTTCTTCACCTTAT
FZD7	GCGCTCATGAACAAGTTCGG	TAGGGCGCGGTAGGGTAG
HEY2	GCCCTCACGGAAAGTTGTGA	TATCCGATCCCGACGCCTTT
HEY1	CGGCTCTAGGTTCCATGTCC	GCTTAGCAGATCCTTGCTCCA
ST3GAL6	AAGGCCATCTGGAGGTAGGG	GAGGCAGGTTGGTTGTCAGT
HEYL	CTGCGTTCGCCATGAAGC	ACGCCGTTTCTCTATGATCCC
SLUG	GGACCACAGTGGCTCAGAAA	GGCCAGCCCAGAAAAAGTTG

Supplementary Table 2. The gene-specific primers used in Chip-qPCR

**Gene**	**Forward Primer 5'-3'**	**Reverse Primer 5'-3'**
FLT4	GAGCCTGGTGGGCAAAG	GAGACTGGGTCTCCCTATGTTA
DLL3	GCCCTGGATCTTCATCGTATC	TGAATGAGACTGGGTTTGAGAG
FZD7	CGAAGATGGGATCCCGAAAG	CTGGTTCTAGAAGTAACCTGAGAC
NOTCH3	ACG AGT CCC GAA CTC TGT A	GGG ACC CTT GAG CGA TTA G
HEY1	ACT CAC TCC TCT CCG TCT TT	TCA GAT CAC CTC TCC TCG TTT A
HEY2	GGG CAA GGG ATG TGG ATT T	GTT TCA GTG GCT GGG ACA T
HEYL	GCC TGA TCC TCA GCA AGA AT	CCT CTC AGA AGT GTA AGT GTC TTC

Supplementary Table 3. The sequence of siRNAs and shRNAs.

Gene name	Sence (5'-3')	**Anti-Sence (5'-3')**
NOTCH3 siRNA 1	CAGCGTGACCGAGATAGGTC A	TGACCTATCTCGGT CACGCTG
NOTCH3 siRNA 2	CCAGTTCACCTGTATCTGTAT	ATACAGATACAGG TGAACTGG
SLUG siRNA 1	GCAUUUGCAGACAGGUCAAd TdT	UUGACCUGUCUG CAAAUGCdTdT
SLUG siRNA 2	TTTAAGGCACCTGAGTTCGCG	CGCGAACTCAGGT GCCTTAAA
NOTCH3 inducible system	GGTGATCGGCTCGGTAGTAAT	

Supplementary Table 4. Clinical pathological characteristics of NPC patients used in discovery cohort for RNA sequencing

Clinical information for discovery cohort	
**Characteristics**	**Patients (n=22)**
**Age**	
<30	2
30-40	6
40-50	11
50-60	2
>60	1
**Tumor size**	
<2mm	3
2-5mm	14
>5mm	5
**Stage (UICC)**	
T stage	
2	2
3	10
4	10
N stage	
1	4
2	9
3	9
M stage	
0	21
1	1
Total stage	
III	4
IV	18
**Chemo-response**	
CR	8
PR	14
**Sex**	
Male	7
Female	15

Supplementary Table 5. Clinical pathological characteristics of NPC patients used in validation cohort

Clinical information for verification cohort	
**Characteristics**	**Patients (n=18)**
**Age**	
<30	5
30-40	2
40-50	6
50-60	3
>60	2
**Tumor size**	
<2mm	1
2-5mm	12
>5mm	5
**Stage (UICC)**	
T stage	
2	0
3	13
4	5
N stage	
1	3
2	9
3	6
M stage	
0	18
1	0
Total stage	
III	8
IV	10
**Chemo-response**	
CR	14
PR	4
**Sex**	
Male	12
Female	6

Supplementary Table 6 Patient demographics and clinical characteristics (NOTCH3)

Characteristic	NOTCH3		P
	Low	High	
Total	53(16.4)	181(57.5)	
**Age, years**			0.382[a]
Median (range)	47(21–67)	48(21–77)	
＞45	21(39.6)	84(46.4)	
≤45	32(60.4）	97(53.6)	
**Sex**			0.221[a]
Female	11(20.8)	53(29.3)	
Male	42(79.2)	128(70.7)	
**WHO Pathologic type**			1.000[b]
I	0(0.0)	1(0.6)	
II	0(0.0)	0(0.0)	
III	53(100)	180(99.4)	
**T stage***			0.606[b]
T1	3(5.7)	5(2.8)	
T2	11(20.8)	31(17.1)	
T3	29(54.7)	101(55.8)	
T4	10(18.9)	44(24.3)	
**N stage***			0.765[a]
N0	6(11.3)	18(9.9)	
N1	12(22.6)	53(29.3)	
N2	22(41.5)	74(40.9)	
N3	13(24.5)	36(19.9)	
**TNM stage***			0.907[b]
I	1(1.9)	4(2.2)	
II	3(5.7)	12(6.6)	
III	30(56.6)	92(50.8)	
IVa	19(35.8)	73(40.3)	
**EBV DNA(copies/mL)**			0.414[a]
<1500	22(41.5)	64(35.4)	
≥1500	31(58.5)	117(64.6)	
**Smoking**			0.166[a]
No	41(77.4)	122(67.4)	
Yes	12(22.6)	59(32.6)	
**LDH concentration(U/L)**			0.421[a]
<245	46(86.8)	164(90.6)	
≥245	7(13.2)	17(9.4)	
**C-reactive protein(mg/L)**			0.500[a]

<3	38(71.7)	138(76.2)	
≥3	15(28.3)	43(23.8)	
**Chemoradiotherapy**			0.435[a]
Yes	50(94.3)	175(96.7)	
No	3(5.7)	6(3.3)	

WHO, World Health Organization; TNM, tumor‑node‑metastases; EBV, Epstein‑Barr virus; LDH, serum lactate dehydrogenase; CRP, serum C reactive protein.

[a]P value was calculated using the chi-square test. [b]P value was calculated using Fisher's exact test.

*According to the 8th edition of UICC/AJCC staging system

Supplementary Table 7 Multivariable analysis of prognostic factors of the NPC patients (NOTCH3)

	**Hazard ratio***	**P value**
**Progression-free survival**		
LDH (≥ 245 *vs*. < 245 U/L)	2.565 (1.130–5.825)	0.024
NOTCH3 expression (high *vs*. low)	2.979 (1.055–8.413)	0.039
**Distant metastasis-free survival**		
CRP (≥ 3 *vs*. < 3 mg/L)	2.487 (1.089–5.679)	0.031
EBV DNA (≥ 1500 *vs*. < 1500copies/mL)	3.508 (1.180–10.431)	0.024
Smoking(yes vs. no)	2.473(1.068-5.725)	0.034
NOTCH3 expression (high *vs*. low)		0.041

Hazard ratios and p-values were calculated using an adjusted multivariate Cox proportional hazards regression model, Notch3 expression (high vs. low), Sex (male vs. female), age (⩾ 45 years vs. <45 years), TNM stage (I–II vs. III-IV), LDH (⩾ 245 vs. < 245 U/L), CRP (⩾ 3 vs. < 3 mg/L), EBV DNA (⩾ 1500 vs. < 1500copies/mL), Smoking(yes vs. no), chemoradiotherapy (yes vs. no) were included as covariates. NPC: nasopharyngeal carcinoma; TNM, tumor–node–metastases; LDH, serum lactate dehydrogenase levels; CRP, serum C-reactive protein level; EBV, Epstein–Barr virus; HR, hazard ratio; CI, confidence interval. Variables were selected with the backward stepwise approach, the p value threshold was 0.1 (p > 0.1) for removing insignificant variables from the model. Only variables significantly associated with survival were presented, marginally significant variables (0.05 < p < 0.1) were remained in the final Cox model but not presented in the table.

Supplementary Table 8 Patient demographics and clinical characteristics (SLUG)

Characteristic	SLUG		P
	High	Low	
**Total**	97(77.6)	28(22.4)	
**Age, years**			0.104[a]
Median (range)	48(28–77)	44.5(25–63)	
＞45	55(56.7)	11(39.3)	
⩽45	42(43.3）	17(60.7)	
**Sex**			0.888[a]
Female	23(23.7)	7(25.0)	
Male	74(76.3)	21(75.0)	
**WHO Pathologic type**			1.000[b]
I	1(1.0)	0(0.0)	
II	0(0.0)	0(0.0)	
III	96(99.0)	28(100)	
**T stage***			0.166[b]
T1	1(1.0)	0(0.0)	
T2	15(15.5)	1(3.6)	
T3	52(53.6)	21(75.0)	
T4	29(29.9)	6(21.4)	
**N stage***			0.386[b]
N0	3(3.1)	0(0.0)	
N1	34(35.1)	6(21.4)	
N2	37(38.1)	12(42.9)	
N3	23(23.7)	10(35.7)	
**TNM stage***			0.166[b]
II	4(4.1)	0(0.0)	
III	48(49.5)	12(42.9)	
IVa	45(46.4)	16(57.1)	
**EBV DNA(copies/mL)**			0.125[b]
<1500	26(26.8)	3(10.7)	
⩾1500	71(73.2)	25(89.3)	
**Smoking**			0.147[a]
No	66(68.0)	23 (82.1)	
Yes	31(32.0)	5(17.9)	
**LDH concentration(U/L)**			0.682[b]
<245	89(91.8)	27(96.4)	
≥245	8(8.2)	1(3.6)	

**C-reactive protein(mg/L)**			0.718[a]
<3	73(75.3)	22(78.6)	
≥3	24(24.7)	6(21.4)	
**Treatment**			NA
Concurrent chemoradiotherapy	97(100)	28(100)	

WHO, World Health Organization; TNM, tumor – node – metastases; EBV, Epstein – Barr virus; LDH, serum lactate dehydrogenase; CRP, serum C reactive protein.

[a]P value was calculated using the chi-square test. [b]P value was calculated using Fisher's exact test.

*According to the 8th edition of UICC/AJCC staging system.

Supplementary Table 9 Multivariable analysis of prognostic factors of the NPC patients (SLUG)

	**Hazard ratio***	***P***
	**(95% CI)**	**value**
**Progression-free survival**		
CRP (≥ 3 *vs*. < 3 mg/L)	2.567 (1.048–6.287)	0. 039
SLUG expression (high *vs*. low)	2.613 (1.067–6.399)	0.036
**Distant metastasis-free survival**		
CRP (≥ 3 *vs*. < 3 mg/L)	4.183 (1.265–13.833)	0.019
TNM stage (I–III *vs*. IV)	6.522 (1.253–28.524)	0.026
Smoking(yes vs. no)	4.954(1.221-20.097)	0.025
SLUG expression (high *vs*. low)	8.914(2.461-32.288)	0.001

Hazard ratios and p-values were calculated using an adjusted multivariate Cox proportional hazards regression model, Notch3 expression (high *vs*. low), Sex (male *vs*. female), age (⩾ 45 years *vs*. <45 years), TNM stage (I–III *vs*. IV), LDH (⩾ 245 *vs*. < 245 U/L), CRP (⩾ 3 *vs*. < 3 mg/L), EBV DNA (⩾ 1500 vs. < 1500copies/mL), Smoking(yes vs. no) were included as covariates. NPC: nasopharyngeal carcinoma; TNM, tumor–node–metastases; LDH, serum lactate dehydrogenase levels; CRP, serum C-reactive protein level; EBV, Epstein–Barr virus; HR, hazard ratio; CI, confidence interval. Variables were selected with the backward stepwise approach, the p value threshold was 0.1 (p > 0.1) for removing insignificant variables from the model. Only variables significantly associated with survival were presented, marginally significant variables (0.05 < p < 0.1) were remained in the final Cox model but not presented in the table.
