# Supplementary material for: Enhancer remodeling activates NOTCH3 signaling to confer chemoresistance in advanced nasopharyngeal carcinoma
Source: Cell Death Dis. 2023 Aug 10;14(8):513. doi: 10.1038/s41419-023-06028-z (PMC10415329; doi:10.1038/s41419-023-06028-z)

Unedited blot for Figure 2

C

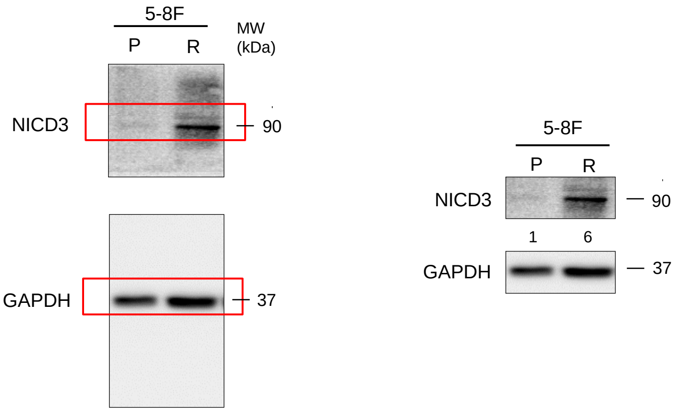

F

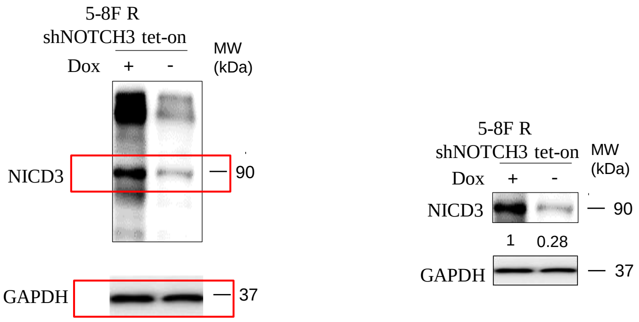

G

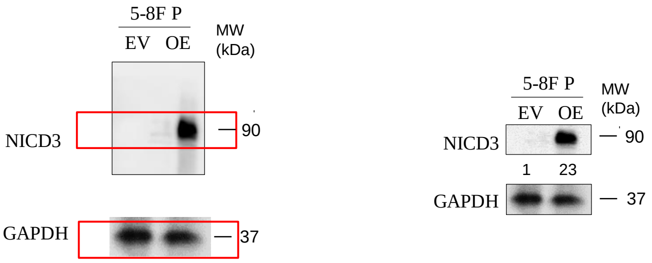

H

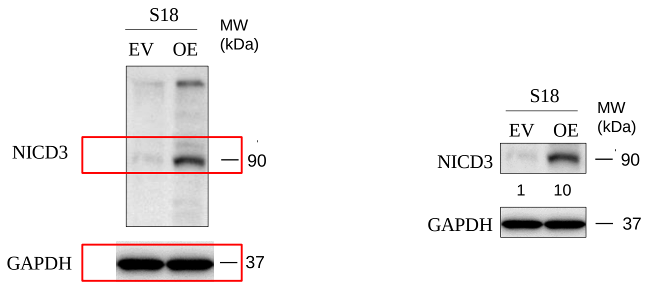

Unedited blot for Figure 4A

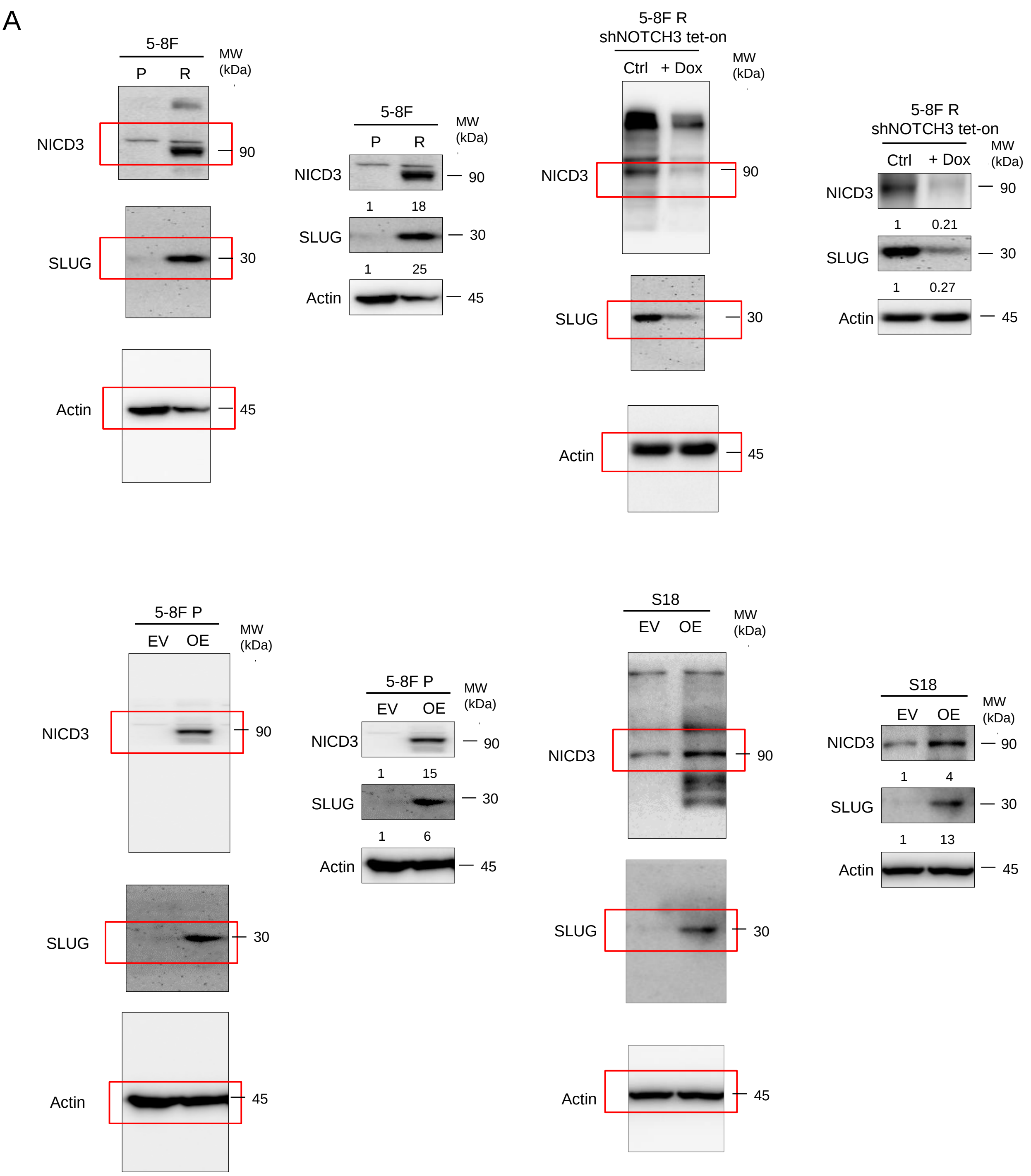

Unedited blot for Figure 4B

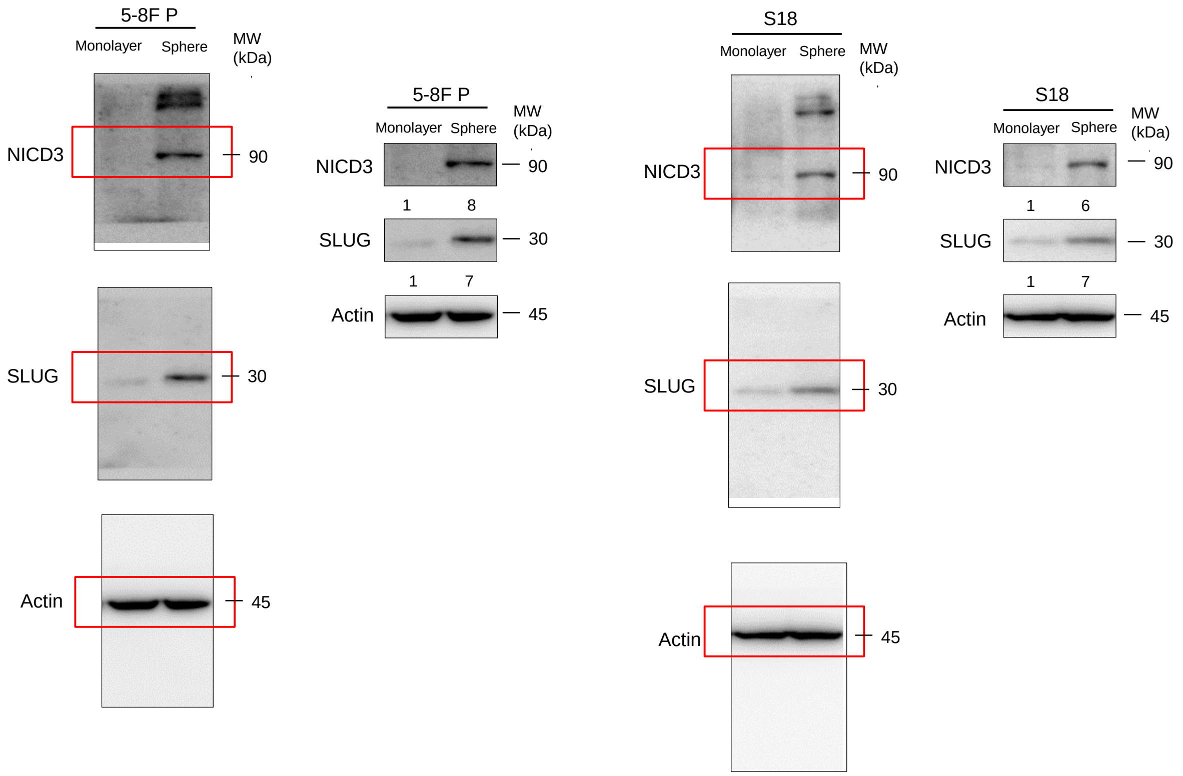

Unedited blot for Figure 4D

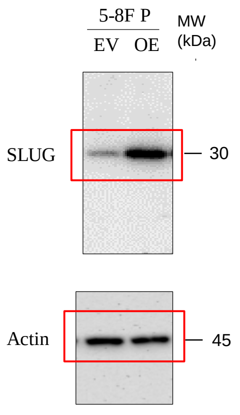

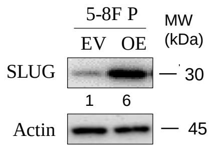

Unedited blot for Figure 5G

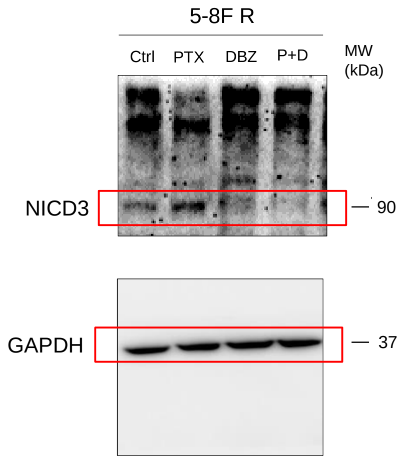

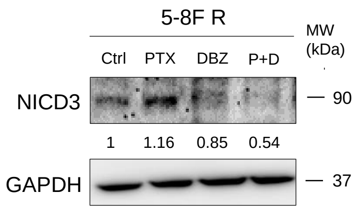

Supplement: Supplementary file 3 — Western uncut [file 41419_2023_6028_MOESM3_ESM.pdf]
